# Readability of Online Hand and Upper Extremity Patient Resources

**DOI:** 10.7759/cureus.36031

**Published:** 2023-03-11

**Authors:** Brian K Foster, Clarice Callahan, C. Liam Dwyer

**Affiliations:** 1 Musculoskeletal Institute, Geisinger Medical Center, Danville, USA; 2 Orthopedic Surgery, Michigan State University College of Human Medicine, Grand Rapids, USA; 3 Orthopedic Surgery, Geisinger Medical Center, Danville, USA

**Keywords:** orthopedic hand surgery, hand surgery, online medical education, online education, patient education, readability

## Abstract

Background

Online patient resources regarding hand and upper extremity topics published by professional societies are written at a level that exceeds that of the average reader.

Methodology

Online patient resources focused on hand and upper extremity topics published by the American Society for Surgery of the Hand (ASSH), the American Association for Hand Surgery (AAHS), and the American Academy of Orthopaedic Surgeons (AAOS) were reviewed. The reading material from each topic page was analyzed using the Flesch-Kincaid Grade Level (FKGL) and Flesch Reading Ease (FRE) formulas. The reading level (FKGL) of each topic page was compared against an eighth-grade reading level, which corresponds to the average US reading level.

Results

A total of 170 online patient resources were reviewed, including 84 from the ASSH, 74 from the AAOS, and 12 from the AAHS. Overall, the mean FKGL was 9.1, and the mean FRE was 57.3. Overall, 50% of all hand and upper extremity online resources were written at or below an eighth-grade reading level. Pairwise testing revealed topic pages written by the ASSH had lower FKGL compared to those written by the AAHS (p = 0.046).

Conclusions

Online patient resources focused on hand and upper extremity topics are, on average, written at a level that exceeds the ability of the average reader. Comparisons between organizations showed a statistical, but not clinical, difference in readability measures. An emphasis on improving readability should be maintained as professional organizations continue to develop their online patient resources.

## Introduction

The internet is a low-cost and easily accessible tool for patients searching for health information. In fact, 67% of orthopedic patients have reported searching for health information online [1]. However, without oversight, the quality of this information remains unknown. Previous studies have demonstrated that the quality of these resources, across areas of orthopedic surgery, is poor [2-6]. Additionally, these resources are often written at a level that is too complex for the average reader [4,5,7]. These deficiencies make it difficult for patients to learn more about their medical problems, which may lead to poor health decisions.

In response, professional societies, such as the American Society for Surgery of the Hand (ASSH) and the American Academy of Orthopaedic Surgeons (AAOS), have created online libraries of patient-directed educational resources. Given their reputation and stature, these sources should represent the highest quality information available online. However, previous studies have demonstrated that these resources are often published at a level that is too complex for the average reader [8-16]. Most recently, in 2016, the average ASSH patient-directed resource was written above the average United States (US) reading level [9].

When high-quality information is not able to be understood, it is not helpful for patients. We aimed to analyze the online patient resources published by the ASSH, AAOS, and American Association for Hand Surgery (AAHS) to investigate any changes in readability level. Additionally, we aimed to compare our findings to previously reported results. In light of the increasing awareness of the deficits of online patient resources, we hypothesized that these resources would be written below the average US reading level.

## Materials and methods

Online patient resources focused on hand and upper extremity topics published by the ASSH (https://www.assh.org/handcare/conditions), AAHS (https://handsurgery.org/public/), and AAOS (https://orthoinfo.aaos.org/en/diseases--conditions/) were reviewed. Readability was assessed using the Flesch-Kincaid Grade Level (FKGL) and Flesch Reading Ease (FRE) formulas. These tools, originally developed for US Navy technical handouts, account for the average number of syllables and number of words in each sentence to determine the difficulty of comprehension. The result of the FKGL equals the corresponding grade level readability. The FRE result ranges from 0 to 100, with higher scores indicating greater readability. After the text was appropriately formatted, as described by Badarudeen and Sabharwal, calculations were performed on Microsoft Word® (Microsoft Corp, Redmond, WA, USA) [17]. The reading level of each topic page was compared against an eighth-grade reading level (defined as FKGL <9), which corresponds to the average US reading level [12].

Statistical analysis

Descriptive statistics were generated for resources’ characteristics such as the professional society, FKGL, and FRE. A one-way analysis of variance (ANOVA) was used to compare differences in FKGL and FRE among the three professional societies. In addition, a chi-square test was used to evaluate the association between the rate of resources at or below eighth-grade reading level and professional society. Furthermore, a post-hoc Tukey-Kramer test and pairwise chi-square comparison were conducted following the one-way ANOVA and chi-square test, respectively, to identify which two groups were statistically significant. All analyses were performed in SAS Enterprise Guide v8.3 (SAS, Inc., Cary, NC, USA), with contrasts of p <0.05 considered statistically significant.

## Results

A total of 170 online patient resources were reviewed, including 84 from the ASSH, 74 from the AAOS, and 12 from the AAHS. The mean FKGL and FRE of all sites were 9.1 (SD = 1.3) and 57.3 (SD = 7.7), respectively. Overall, 50% (n = 85) of all topic pages had FKGL less than 9.

Table 1 displays a comparison of resource characteristics among the three professional societies. The average FKGL for the ASSH, AAHS, and AAOS was 8.9 (SD = 1.3), 9.9 (SD = 1.8), and 9.3 (SD = 1.1), respectively. One-way ANOVA demonstrated a significant difference in FKGL and FRE between at least two professional societies. The post-hoc Tukey-Kramer test revealed that the difference in FKGL between ASSH and AAHS was significant (p = 0.046, Table 2).

**Table 1:** Comparison of resource characteristics among professional societies. ^a^: Significant association (p = 0.03) when compared against ASSH.

Characteristic	American Association for Hand Surgery (AAHS), N = 12 (7.1%)	American Academy of Orthopaedic Surgeons (AAOS), N = 74 (43.5%)	American Society for Surgery of the Hand (ASSH), N = 84 (49.4%)	P-value
Flesch-Kincaid Grade Level, mean (SD)	9.9 (1.8)	9.3 (1.1)	8.9 (1.3)	0.03
Flesch Reading Ease, mean (SD)	54.1 (9.9)	55.6 (6.9)	59.3 (7.6)	0.003
At/Below eighth-grade reading level, n (%)				
Yes	3 (25.0%)^a^	33 (44.6%)	49 (58.3%)	0.045

**Table 2:** Post-hoc Tukey-Kramer comparisons. ASSH = American Society for Surgery of the Hand; AAOS = American Association of Orthopaedic Surgeons; AAHS = American Association for Hand Surgery

Characteristic	Pairwise Group		Difference in means (95% CI)	P-value
Flesch-Kincaid Grade level	AAHS	AAOS	0.573 (-0.372, 1.518)	0.33
	ASSH	AAHS	-0.950 (-1.887, -0.013)	0.046
	ASSH	AAOS	-0.377 (-0.861, 0.107)	0.16
Flesch Reading Ease	AAHS	AAOS	-1.514 (-7.032, 4.004)	0.79
	ASSH	AAHS	5.196 (-0.276, 10.669)	0.1
	ASSH	AAOS	-3.682 (-6.509, -0.855)	0.01

The rate of topic pages written at or below an eighth-grade level by the ASSH, AAHS, and AAOS was 58.3%, 25%, and 44.6%, respectively. One-way ANOVA demonstrated a significant difference in this rate between at least two professional societies. Post-hoc chi-square comparison revealed a significant difference between ASSH and AAHS (p = 0.03).

## Discussion

In this review of online patient resources on hand and upper extremity topics, the readability of resources published by professional societies is still too complex for the average reader. Half of all topic pages reviewed exceeded the reading level of the average US patient. Additionally, there was a statistically significant difference in readability scores between the different organizations. However, the absolute differences in scores are so small that these differences are likely not clinically meaningful.

Previous studies investigating patient resources published by the ASSH, AAHS, and AAOS have raised concerns about their readability (Table 3). Wang et al. first evaluated the ASSH and AAOS hand and wrist topic pages, demonstrating an average FKGL of 10.5 and 8.5, respectively [8]. Yi et al. assessed resources by the AAHS and reported an average FKGL of 8.98 [14]. In a follow-up study several years later, Hadden et al. showed improvement in ASSH resource readability, as sites scored a mean FKGL of 9.3 [9]. Our results demonstrate that the readability of ASSH resources continues to improve, whereas resources from the AAOS and AAHS have remained unchanged, or even regressed.

**Table 3:** Reported grade-level readability of upper extremity resources over time. NR = not reported; ASSH = American Society for Surgery of the Hand; AAOS = American Association of Orthopaedic Surgeons; AAHS = American Association for Hand Surgery

Author	Year published	Flesch-Kincaid Grade Level score, mean (SD)		
		ASSH	AAHS	AAOS
Wang et al. [8]	2009	10.4 (1.6)		8.5 (1.0)
Beutel et al. [13]	2013	10.3 (1.7)		9.4 (1.5)
Yi et al. [14]	2014		8.98 (NR)	
Hadden et al. [9]	2016	9.3 (NR)		
Roberts et al. [11]	2016			8.7 (1.1)
Present study	2021	8.9 (1.3)	9.9 (1.8)	9.3 (1.1)

Our results highlight the issue of readability of patient-directed information, which is pervasive across orthopedics and includes all subspecialty professional organizations [10-12,15,16]. Roberts et al. reviewed 435 AAOS topic pages, finding an average FKGL of 9.3 and noting 84% of pages were written above an eighth-grade reading level [11]. These poor results speak to the immense difficulty of creating high-quality and informative resources for a broad audience of varying health literacy and on topics that are inherently complex. This is compounded by the issue that musculoskeletal health literacy lags behind general health literacy [18]. Professional societies, having the innate trust of patients for providing high-quality information, should strive to reach as large an audience as possible by improving the readability of their resources.

There are several limitations that must be considered when interpreting these results. First, there are innate limitations to using readability formulas that fail to consider the meaning of each included word. The FKGL and FRE, instead, use word and sentence lengths as a surrogate for language complexity. Roberts et al. recently questioned the external validity of these formulas as scores did not correlate with patients’ self-reported understanding of educational materials [19]. However, to effectively compare changes over time, we mirrored our methods to previous studies. Additionally, these tools are not able to account for the added value of pictures or videos in the understanding of these resources.

## Conclusions

Online patient-directed resources on hand and upper extremity-related topics continue to be written at a level that exceeds the ability of the average reader. Comparisons between organizations showed a statistically significant, but not clinically meaningful, difference in readability measures. An emphasis on improving readability should be maintained as professional organizations continue to develop their online patient resources.
